# Therapeutic strategy for the patients with coexisting gastroesophageal reflux disease and postprandial distress syndrome of functional dyspepsia

**DOI:** 10.1002/jgh3.12299

**Published:** 2020-02-03

**Authors:** Kimio Isshi, Hiroto Furuhashi, Koji Nakada

**Affiliations:** ^1^ Department of Gastoroenterology, Isshi Gastro‐Intestinal Clinic Tokyo Japan; ^2^ Department of Endoscopy Jikei University School of Medicine Tokyo Japan; ^3^ Department of Laboratory Medicine The Jikei University Daisan Hospital Tokyo Japan

**Keywords:** acotiamide, combined therapy, functional dyspepsia, gastroesophageal reflux disease, proton pump inhibitor

## Abstract

**Background and Aim:**

Gastroesophageal reflux disease (GERD) and functional dyspepsia (FD) frequently overlap. However, no accepted treatment has yet been established for such patients. This study was conducted to identify an adequate initial treatment for patients with GERD accompanied by the postprandial distress syndrome type of FD (FD‐PDS).

**Methods:**

Of the 150 patients newly diagnosed with GERD who visited our clinic, 53 patients with the typical symptoms of both GERD and FD‐PDS were assessed using the modified frequency scale for the symptoms of GERD and the gastroesophageal reflux and dyspepsia therapeutic efficacy and satisfaction test questionnaires. Of those, 42 patients who completed 4 weeks of treatment were analyzed. We compared the treatment responses between the 21 patients who received proton pump inhibitor (PPI) monotherapy and 21 patients who received a PPI in combination with the prokinetic drug acotiamide.

**Results:**

Assessment of the two questionnaires revealed a marked improvement of both GERD and FD symptom scores after 4 weeks of treatment in both groups. However, there were no significant differences in any GERD or FD symptom scores at baseline, after 4 weeks of treatment and in the symptom score change between the two different treatment groups.

**Conclusion:**

The results of this retrospective study suggest no benefit of PPI‐prokinetic combination *versus* PPI monotherapy in adult patients with FD‐GERD overlap; therefore, PPI monotherapy could be an adequate initial treatment for such patients.

## Graphical abstract

There was no clinical benefit evident by adding acotiamide to the proton pump inhibitor (PPI) regimen as an initial treatment for the patients with coexisting gastroesophageal reflux disease and postprandial distress syndrome type of FD symptoms; therefore, PPI monotherapy could be an appropriate choice for initial treatment in such patients.

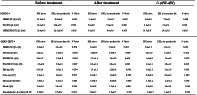

## Introduction

Gastroesophageal reflux disease (GERD) and functional dyspepsia (FD) are frequently encountered in routine clinical practice. Furthermore, GERD symptoms are frequently known to coexist with the symptoms of FD,^1^, ^2^, ^3^, ^4^ and proton pump inhibitors (PPIs) and prokinetic drugs are often coadministered in patients with coexisting GERD and FD symptoms in clinical practice. However, no effective initial treatment has been identified for patients with coexisting symptoms of GERD and postprandial distress syndrome, which is a type of functional dyspepsia (FD‐PDS).

Japanese clinical practice guidelines for GERD recommend the use of a PPI as the first‐line agent for the treatment of GERD.^5^ On the other hand, Japanese clinical practice guidelines for FD propose selection of the initial therapeutic agent according to the type of FD: acid secretion inhibitory drugs are recommended for epigastric pain syndrome (EPS), while prokinetic drugs are recommended for PDS.^6^ Acotiamide is the only prokinetic agent that is covered by health insurance in Japan for the disease named FD according to insurance nomenclature, and it has been reported to be effective against FD‐PDS.^7^ Therefore, we hypothesized that combined therapy with a PPI and acotiamide may be more effective than a PPI alone in patients with coexisting GERD and FD‐PDS symptoms.

In this study, we compared the therapeutic responses of 4 weeks' treatment with a PPI alone with that of 4 weeks' combined therapy with a PPI plus acotiamide in patients with coexisting GERD and FD‐PDS symptoms, to identify the appropriate initial treatment for patients presenting with an overlap of GERD and FD.

## Methods

### *Study design*

This study was a single‐center, retrospective study conducted in accordance with the principles of the Declaration of Helsinki (sixth revision 2013), with the approval of the ethics committee of the Japan Medical association Ethical Review Board, Tokyo, Japan.

### *Patients*

Of the 150 patients newly diagnosed with GERD who visited our clinic, 53 patients with coexisting GERD and FD‐PDS symptoms were enrolled. An upper gastrointestinal endoscopy was performed in all patients and those with any organic lesions other than reflux esophagitis were excluded from this study. Two symptom‐based questionnaires, the modified frequency scale for the symptoms of GERD (MFSSG)^8^ and the gastroesophageal reflux and dyspepsia therapeutic efficacy and satisfaction test (GERD‐TEST)^9^, ^10^ were completed by the patients at prior to treatment and after 4 weeks of treatment as a routine in our clinic. The patients were treated with alternating by either a PPI alone at the usual dose (20 mg of esomeprazole or 10 mg of rabeprazole as a single daily dose at bedtime) or a PPI in combination with acotiamide (300 mg administered in 3 divided doses, before meals) for 4 weeks. Daily dietary and lifestyle guidance were provided for all cases.

### *Data collection*

The severity of the reflux esophagitis was assessed by upper gastrointestinal endoscopy according to the modified Los Angeles classification system. Patient characteristics (gender, age, body mass index [BMI]) were collected from the medical records. The severity of the GERD and dyspeptic symptoms and the burden on their daily living status caused by symptoms were assessed using the symptom‐based questionnaires, the MFSSG, and the GERD‐TEST, prior to treatment and after 4 weeks of treatment.

### *Questionnaires for data collection*

MFSSG is a modification of the frequency scale for the symptoms of GERD (FSSG),^11^ which was widely used in questionnaires for GERD, modified to utilize it also for FD patients by adding two FD‐related symptoms, epigastric pain at fasting and after meal. As a result, the MFSSG consists of 14 items (i.e. 7 GERD‐related and 7 FD‐related symptom items). The response to each item is rated on a five‐point symptom frequency scale (Table 1). The GERD‐TEST has developed and validated by the study committee of the GERD society and is an official questionnaire of the GERD Society, a Japanese collaborative research group consisting of experts in the clinical practice of GERD. The GERD‐TEST is easy to understand as it consists of a minimal number of items. The GERD‐TEST is composed of 13 items for investigating GERD and dyspepsia symptoms, the impact of the symptoms on the patients' daily living status, and the patients' impression about the effect of the therapy. Questions Q1–Q5 of the GERD‐TEST are for assessing the severity of the upper abdominal symptoms; Q6–Q9 are for assessing the impact of the symptoms on the daily living status of the patients, including eating, sleeping, daily activities, and mood; Q10–Q12 are for evaluating the therapeutic responses to treatment; Q13 is to determine the patient's compliance with the prescribed medication; the responses to Q1–Q11 and Q13 are graded on a Likert scale, while those to Q12 are graded on a numerical rating scale (NRS) (Table 2).

**Table 1:** Modified frequency scale for the symptoms of gastroesophageal reflux disease (MFSSG)

	Circle the appropriate response				
Question	Never	Occasionally	Sometimes	Often	Always
1. Do you get heartburn?	0	1	2	3	4
2. Do you sometimes subconsciously rub your chest with your hand?	0	1	2	3	4
3. Do you get heartburn after meals?	0	1	2	3	4
4. Does something get stuck when you swallow?	0	1	2	3	4
5. Do you get bitter liquid(acid) coming up into your throat?	0	1	2	3	4
6. Do you get heartburn if bend over?	0	1	2	3	4
7. Do you have an usual (e.g. burning) sensation in your throat?	0	1	2	3	4
8. Does your stomach get bloated?	0	1	2	3	4
9. Does your stomach ever feel heavy after meals?	0	1	2	3	4
10. Do you ever feel sick after meals?	0	1	2	3	4
11. Do you feel full while eating meals?	0	1	2	3	4
12. Do you burp a lot?	0	1	2	3	4
13. Do you get epigastric pain (burning) after meals?	0	1	2	3	4
14. Do you get epigastric pain (burning) before meals?	0	1	2	3	4

**Table 2:** Gastroesophageal reflux and dyspepsia therapeutic efficacy and satisfaction test (GERD‐TEST)

Q1. Have you been bothered by heartburn during the past week? (By heartburn we mean a burning pain or discomfort behind the breastbone in your chest)
Q2. Have you been bothered by acid regurgitation during the past week? (By acid regurgitation we mean regurgitation or flow of sour or bitter fluid into your mouth)
Q3. Have you been bothered by epigastric pain or burning during the past week? (Epigastric pain includes any type of pain of the stomach)
Q4. Have you been bothered by postprandial fullness during the past week? (Postprandial fullness refers to discomfort or a sensation of heaviness caused by the food you consume remaining in the stomach)
Q5. Have you been bothered by early satiation during the past week? (Early satiation refers to the inability to finish a normally sized meal)
Response scale for Q1–Q5:
1 = no discomfort at all, 2 = slight discomfort, 3 = mild discomfort, 4 = moderate discomfort, 5 = moderately severe discomfort, 6 = severe discomfort, 7 = very severe discomfort.
Q6. During the past week, how often have you felt dissatisfaction because you were unable to eat meals as you intended due to chest and stomach symptoms? (Not being able to eat as you intended refers to the inability to eat the sufficient amount of food you want to eat at an uninhibited, natural pace)
Q7. During the past week, how often have you felt dissatisfaction due to impaired sleep caused by chest and stomach symptoms?
Q8. During the past week, how often have you felt dissatisfaction due to impairment of your work, housework, or other daily activities caused by chest and stomach symptoms?
Q9. During the past week, how often have you felt dissatisfaction because you were in a bad mood due to chest and stomach symptoms?
Response scale for Q6–Q9:
1 = not at all, 2 = slightly, 3 = moderately, 4 = quite a lot, 5 = extremely
Q10. During the past week, how often have you wanted another drug in addition to the drug your doctor prescribed because of intense symptoms of heartburn and acid regurgitation?
1 = not at all, 2 = on 1 day, 3 = on 2 to 3 days, 4 = on 4 to 5 days, 5 = always.
Q11. During the past week, how have you felt about symptoms of heartburn and acid regurgitation as compared with the symptom severity before current treatment?
1 = extremely improved, 2 = improved, 3 = slightly improved, 4 = not changed, 5 = aggravated.
Q12. If 10 corresponds to your symptoms before current treatment and 0 is “symptom‐free,” what number corresponds to symptoms of heartburn and acid regurgitation during the past week? Please circle the applicable score below:
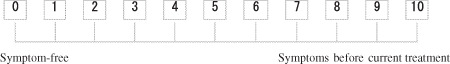
Q13. What proportion of the proton pump inhibitor prescribed to you did you take as instructed?
1 = took drug as instructed, 2 = generally took drug as instructed (took at least three‐quarters of the drug prescribed), 3 = sometimes forgot (took at least half but less than three‐quarters of the drug prescribed, 4 = took little (took less than half of the drug prescribed), 5 = did not take any.

Note: Before therapy, questions about treatment efficacy and adherence (Q10–Q13) were excluded. The following scores were defined: GERD symptom score = (Q1 + Q2)/2, Epigastric pain/burning symptoms score = Q3, Postprandial distress symptom subscale = (Q4 + Q5)/2, Residual symptom rate (%) = 100 × (GERD symptom score at 4 weeks − 1)/(GERD symptom score at 0 week − 1).

### *Definitions of the scores on the GERD TEST*

The GERD symptom subscale (SS) score was calculated as the mean of the scores for GERD‐TEST Q1 (heartburn) and Q2 (acid regurgitation). The EPS symptom score was defined as the score for Q3, the PDS‐SS score was calculated as the mean of the scores for Q4 and Q5, and the FD‐SS score was calculated as the mean of the EPS symptom score and PDS‐SS score. The dissatisfaction for daily living status‐SS score was calculated as the mean of the scores for Q6 (eating), Q7 (sleeping), Q8 (daily activity), and Q9 (mood).

### *Outcome measures*

To investigate the therapeutic responses to treatment with a PPI alone and combined treatment with a PPI plus acotiamide, the scores on the MFSSG (i.e. the sum of the total scores for GERD‐related symptoms [GERD‐TS], FD‐related symptoms [FD‐TS] and overall symptoms [GERD/FD‐TS]), scores on the GERD‐TEST (i.e. the GERD‐SS, FD‐EPS‐symptom [Sx], FD‐PDS‐SS and FD‐SS), and the impact of the symptoms on the daily living status of the patients (i.e. scores for the questions related to eating, sleeping, daily activity, mood, and the dissatisfaction for daily life‐SS) were compared between that prior to treatment and after 4 weeks of treatment in each group. Then, to compare the therapeutic responses of treatment with a PPI alone and combined treatment with a PPI plus acotiamide, the aforementioned scores before and after 4 weeks of treatment and the changes in the scores after 4 weeks of treatment against the pre‐treatment scores were compared between the PPI alone and PPI plus acotiamide groups.

### *Statistical analysis*

The data of the patients who underwent the baseline endoscopic examination, answered the two questionnaires at 0 week and 4 weeks, provided responses to at least questions about the gender, age, and BMI, Q1–Q14 of the MFSSG, and Q1–Q9 of the GERD‐TEST, and showed a medication adherence rate of at least 75% were used to analyze the responses to the treatment.

To examine the responses to PPI monotherapy and the combination therapy with PPI and acotiamide, the symptom and daily living status scores before and after therapy were compared using a paired *t*‐test. To compare the therapeutic responses between the PPI alone and the PPI plus acotiamide groups, the scores on the MFSSG and the GERD‐TEST before treatment and after 4 weeks of treatment, and the changes in the scores after the treatment were compared using unpaired *t*‐tests. The effect sizes (Cohen's *d*) were also calculated. Cohen's *d* values of ≧0.20, ≧0.50 and ≧0.80 were considered to represent small, medium, and large effects, respectively. Data analysis was performed using the JMP12.0.1 software (SAS Institute Inc., Cary, NC, USA). All statistical tests were performed using a two‐sided test, with the significance level set at *P* < 0.05.

## Results

### *Patient characteristics*

The subjects consisted of 21 patients in the PPI alone group and 21 patients in the PPI plus acotiamide group. The mean ages (mean ± SD) were 50.8 ± 16.8 and 49.1 ± 17.0 years and the mean BMI values (mean ± SD) were 22.8 ± 4.4 and 22.7 ± 2.9 kg/m^2^ in the PPI alone and PPI plus acotiamide groups, respectively; the male/female ratios were 4/17 and 8/13, the frequency ratios of the endoscopic findings of erosive reflux disease (ERD)/non‐erosive reflux disease (NERD) were 1/20 and 4/17, and frequency ratios of EPS + PDS/PDS (types of FD) were 12/8 and 10/11 in the two groups, respectively. There were no significant differences in any of the other patient characteristics between the two groups (Table 3).

**Table 3:** Patients' characteristics

	PPI alone group (*n* = 21)	PPI plus acotiamide group (*n* = 21)	*P* value
Age (year)	50.8 ± 16.8	49.1 ± 17.0	0.744†
BMI	22.8 ± 4.4	22.7 ± 2.9	0.933†
Gender			
Male/female	4/17	8/13	0.172‡
Endoscopic findings?			
ERD/NERD	1/20	4/17	0.153‡
Type of FD			
EPS + PDS/PDS	12/8	10/11	0.427‡

† *t*‐test.

‡ Chi‐square test.

Data are presented as means ± SD.

BMI, body mass index (kg/m^2^); EPS, epigastric pain syndrome; ERD, erosive reflux disease; FD, functional dyspepsia; NERD, non‐erosive reflux disease; PDS, postprandial distress syndrome; PPI, proton pump inhibitor.

### *Therapeutic responses of the protocol treatments against the GERD/FD symptoms*

Assessment by the MFSSG revealed marked improvement of the GERD‐TS, FD‐TS, and GERD/FD‐TS after 4 weeks of treatment in both the PPI alone and PPI plus acotiamide groups. Cohen's *d* effect sizes ranged from 1.24 to 2.14, indicating large effects.

Assessment by the GERD‐TEST revealed marked improvement of the GERD‐SS, FD‐EPS‐Sx, FD‐PDS‐SS, and FD‐SS in both the PPI alone and PPI plus acotiamide groups. Cohen's *d* effect sizes were 1.14–2.54, indicating large effects, except for a medium effect size for the FD‐EPS‐Sx in the PPI plus acotiamide group (0.74) (Table 4).

**Table 4:** Therapeutic responses of the protocol treatments against the GERD/FD symptoms and on the impact of these symptoms on the daily living status of the patients

	PPI alone group				PPI plus acotiamide group			
MFSSG†	Before Tx (0 week)	After Tx (4 weeks)	*P* value	Cohen's *d*	Before Tx (0 week)	After Tx (4 weeks)	*P* value	Cohen's *d*
GERD‐TS (Q1–Q7)	11.7 ± 4.5	3.1 ± 3.8	<0.0001	2.14	10.3 ± 5.8	3.1 ± 3.7	<0.0001	1.52
FD‐TS (Q8–Q14)	11.3 ± 5.0	5.2 ± 4.5	<0.0001	1.31	12.4 ± 6.7	5.3 ± 4.9	<0.0001	1.24
GERD/FD‐TS (Q1–Q14)	23.0 ± 8.3	8.3 ± 7.6	<0.0001	1.90	22.7 ± 10.0	8.4 ± 7.9	<0.0001	1.62
GERD‐TEST†	Before Tx (0 week)	After Tx (4 weeks)	*P* value	Cohen's *d*	Before Tx (0 week)	After Tx (4 weeks)	*P* value	Cohen's *d*
GERD‐SS (Q1, Q2)	3.6 ± 0.8	1.6 ± 0.8	<0.0001	2.54	3.4 ± 1.2	1.7 ± 0.8	<0.0001	1.76
FD‐SS (Q3–Q5)	3.3 ± 1.1	1.9 ± 0.9	<0.0001	1.42	3.3 ± 1.4	1.9 ± 0.9	<0.0001	1.14
FD‐EPS‐Sx (Q3)	3.3 ± 1.7	1.7 ± 1.1	<0.0001	1.15	3.0 ± 1.9	1.9 ± 1.0	<0.0001	0.74
FD‐PDS‐SS (Q4,Q5)	3.3 ± 0.8	2.0 ± 1.2	<0.0001	1.29	3.5 ± 1.3	2.0 ± 1.1	<0.0001	1.30
Eating (Q6)	2.7 ± 1.4	1.7 ± 1.1	0.012	0.83	2.9 ± 1.1	1.5 ± 0.8	<0.0001	1.52
Sleeping (Q7)	1.9 ± 1.3	1.3 ± 0.7	0.063	0.60	1.9 ± 1.3	1.2 ± 0.5	0.053	0.63
Daily activity (Q8)	2.9 ± 1.5	1.6 ± 0.8	0.001	1.15	2.7 ± 1.2	1.5 ± 0.8	0.001	1.17
Mood (Q9)	3.4 ± 1.0	1.7 ± 0.8	<0.0001	1.98	3.3 ± 1.1	1.7 ± 1.0	<0.0001	1.60
Dissatisfaction for daily life‐SS	2.7 ± 0.7	1.5 ± 0.7	<0.0001	1.46	2.7 ± 0.9	1.5 ± 0.7	<0.0001	1.53

† Data are presented as mean ± SD.

EPS, epigastric pain syndrome; FD, functional dyspepsia; GERD, gastroesophageal reflux disease; GERD‐TEST, GERD and dyspepsia therapeutic efficacy and satisfaction test; MFSSG, modified frequency scale for the symptoms of GERD; PDS, postprandial distress syndrome; PPI, proton pump inhibitor; SS, symptom subscale; Sx, symptom; TS, total score; Tx, treatment.

### *Therapeutic responses of the protocol treatments against the impact of the symptoms on the daily living status of the patients*

Marked improvement of the scores showing the impact of the symptoms on the daily living status of the patients (scores for the questions related to dissatisfaction in eating, daily life activities, and mood and the dissatisfaction for daily living status‐SS, but not the score for the question related to dissatisfaction in sleeping) were observed in both the PPI alone and PPI plus acotiamide groups, with large effect sizes (Cohen's *d* values, 1.98–0.83). However, the score for the question related to dissatisfaction in sleeping showed only marginal improvement, with medium effect sizes in both the PPI alone and PPI plus acotiamide groups (*P* = 0.063, Cohen's *d* = 0.60 and *P* = 0.053, Cohen's *d* = 0.63, respectively) (Table 4).

### *Comparison of the treatment responses between the PPI alone and PPI plus acotiamide groups*

In regard to the effects on the MFSSG scores, there were no significant differences in the GERD‐TS, FD‐TS or GERD/FD‐TS at before or after therapy or in the changes of these scores after 4 weeks of treatment (0 week–4 weeks) between the PPI alone and PPI plus acotiamide groups (Table 3). In regard to the effects on the GERD‐TEST scores, there were no significant differences in GERD‐SS, FD‐EPS‐Sx, FD‐PDS‐SS or FD‐SS at before or after the therapy or in the changes of these scores after 4 weeks of treatment (0 week–4 weeks) between the PPI alone and PPI plus acotiamide groups (Table 5). In addition, there were no significant differences in the scores reflecting the impact of the symptoms on the daily living status of the patients (the scores for questions related to dissatisfaction in eating, sleeping, daily life activities, and mood, and the dissatisfaction for daily life‐SS, which integrates these items) before or after therapy, or in the changes of these scores after 4 weeks of treatment, between the PPI alone and PPI plus acotiamide groups (Table 5).

**Table 5:** Comparison of the treatment responses against the GERD/FD symptoms and on the impact of these symptoms on the daily living status of the patients between the PPI alone and PPI plus acotiamide groups

	Before treatment			After treatment			Δ(0 W‐4 W)		
MFSSG †	PPI alone	PPI plus acotiamide	*P* value	PPI alone	PPI plus acotiamide	*P* value	PPI alone	PPI plus acotiamide	*P* value
GERD‐TS (Q1–Q7)	11.7 ± 4.5	10.3 ± 5.8	0.391	3.1 ± 3.8	3.1 ± 3.7	0.967	8.6 ± 3.9	7.2 ± 4.1	0.258
FD‐TS (Q8–Q14)	11.3 ± 4.5	12.4 ± 6.7	0.551	5.2 ± 4.5	5.3 ± 4.9	0.948	6.1 ± 3.0	7.1 ± 5.1	0.440
GERD/FD‐TS (Q1–Q14)	23.0 ± 8.3	22.7 ± 10.0	0.920	8.3 ± 7.6	8.4 ± 7.9	0.953	14.7 ± 5.6	14.3 ± 7.5	0.835
GERD‐TEST†	PPI alone	PPI plus acotiamide	*P* value	PPI alone	PPI plus acotiamide	*P* value	PPI alone	PPI plus acotiamide	*P* value
GERD‐SS (Q1, Q2)	3.6 ± 0.8	3.4 ± 1.2	0.458	1.6 ± 0.8	1.7 ± 0.8	0.843	2.0 ± 1.1	1.7 ± 1.3	0.463
FD‐SS (Q3–Q5)	3.3 ± 1.1	3.3 ± 1.4	0.951	1.9 ± 0.9	1.9 ± 0.9	0.805	1.4 ± 1.0	1.3 ± 1.0	0.762
FD‐EPS‐Sx (Q3)	3.3 ± 1.7	3.3 ± 1.9	0.551	1.7 ± 1.1	1.9 ± 1.0	0.649	1.6 ± 1.5	1.1 ± 1.3	0.279
FD‐PDS‐SS (Q4, Q5)	3.3 ± 0.8	3.5 ± 1.3	0.407	2.0 ± 1.2	2.0 ± 1.1	1.000	1.3 ± 1.1	1.5 ± 1.3	0.446
Eating (Q6)	2.7 ± 1.4	2.9 ± 1.1	0.534	1.7 ± 1.1	1.5 ± 0.8	0.530	1.0 ± 1.2	1.4 ± 1.0	0.218
Sleeping (Q7)	1.9 ± 1.3	1.9 ± 1.3	0.907	1.3 ± 0.7	1.2 ± 0.5	0.809	0.6 ± 0.9	0.6 ± 1.3	1.000
Daily activity (Q8)	2.9 ± 1.3	2.7 ± 1.2	0.653	1.6 ± 0.8	1.5 ± 0.8	0.850	1.3 ± 1.2	1.2 ± 1.2	0.703
Mood (Q9)	3.4 ± 1.0	3.3 ± 1.1	0.768	1.7 ± 0.8	1.7 ± 1.0	1.000	1.7 ± 1.0	1.6 ± 1.0	0.763
Dissatisfaction for daily life‐SS	2.7 ± 0.9	2.7 ± 0.9	0.933	1.5 ± 0.7	1.5 ± 0.7	0.747	1.2 ± 0.7	1.2 ± 0.7	0.823

† Data are presented as mean ± SD.

Δ, change in the score by the treatment; EPS, epigastric pain syndrome; FD, functional dyspepsia, GERD, gastroesophageal reflux disease; GERD‐TEST, GERD and dyspepsia therapeutic efficacy and satisfaction test; MFSSG, modified frequency scale for the symptoms of GERD; PDS, postprandial distress syndrome; PPI, proton pump inhibitor; SS, symptom subscale, Sx, symptom; TS, total score; Tx, treatment.

## Discussion

GERD and FD are commonly encountered in daily clinical practice, and the two diseases frequently overlaps. Patients with coexisting GERD and FD symptoms commonly receive combined treatment with a PPI and prokinetic drug at primary care clinics, although there is insufficient evidence of the usefulness of prescribing combined therapy as the initial treatment. In the present study, we examined the treatment responses of a PPI administered alone and of PPI administered in combination with acotiamide, a prokinetic drug, in patients with coexisting GERD and FD‐PDS symptoms, and found marked improvement of both the GERD and FD symptoms in both the treatment groups. In contrast, there were no significant differences in the treatment responses against the GERD or FD symptoms between the two groups. These results may suggest the helpless of the concomitant administration of acotiamide with a PPI. This is the first report to on comparison of the therapeutic responses between PPI monotherapy and combined therapy with a PPI and prokinetic drug as the initial treatment in patients with coexisting GERD and FD‐PDS symptoms.

Impairment in the quality of life (QOL) is known in both patients with GERD and those with FD, and coexistence of the symptoms of both GERD and FD may be associated with a further deterioration in the QOL.^3^ Therefore, it is important to explore and identify effective treatment for patients with coexisting GERD and FD symptoms.

The Japanese clinical practice guidelines for GERD recommend PPIs as the first‐line agents for the treatment of GERD.^5^ GERD is considered as an acid‐related disease, and treatment with a PPI has been shown to be highly effective.^12^, ^13^

Mainly acid secretion inhibitory and prokinetic drugs are used to treat FD, and meta‐analyses have shown that both are effective.^14^, ^15^ Studies comparing the symptom types of FD have suggested that EPS symptoms are gastric acid‐related, and that acid secretion inhibitory drugs, including PPIs, are effective against EPS symptoms^16^, ^17^; on the other hand, decreased gastric motility, such as impaired gastric accommodation^18^, ^19^ and delayed gastric emptying,^20^, ^21^ is thought to be involved in the development of PDS symptoms, and prokinetic drugs have been reported to be effective against these symptoms.^14^, ^15^, ^22^, ^23^ The Japanese clinical practice guidelines for FD recommend prescribing acid secretion inhibitory drugs for FD‐EPS symptoms and prescribing prokinetic drugs for PDS symptoms.^6^

Acotiamide is a new prokinetic drug and the only therapeutic agent for FD covered by health insurance in Japan. Randomized controlled trials have demonstrated the efficacy of acotiamide against FD‐PDS symptoms.^7^, ^19^, ^24^

Although combined treatment with a PPI and prokinetic drug is often used as the initial treatment for patients with coexisting GERD and FD‐PDS symptoms at primary care clinics, the advantage of such combination therapy has not yet been conclusively demonstrated.

Based on the above, we hypothesized that combined use of PPIs with acotiamide may increase the therapeutic responses in patients with coexisting GERD and FD‐PDS symptoms, and then, compared the therapeutic responses of a PPI administered alone with that of combined therapy with a PPI and acotiamide. However, against our expectations, that the therapeutic responses on either GERD or FD symptoms was equivalent between the PPI alone and combined PPI plus acotiamide treatment groups even as to effect size, Cohen's *d*. Since the concomitantly used acotiamide with a PPI seems helpless, PPI monotherapy is rather recommended as a first‐line treatment for patients coexisting with GERD and FD‐PDS.

Although both the PPI monotherapy and combined PPI plus acotiamide treatment markedly improved GERD and FD symptoms, the beneficial effect on the dissatisfaction with eating was approximately two‐fold stronger in the combination therapy group compared with the monotherapy group as to the effect size, Cohen's *d* (1.52 *vs* 0.83) (Table 2). This may be, in part, explained by the prokinetic effect of acotiamide on the stomach like ameliorating impaired accommodation or delayed gastric emptying.

A recent meta‐analysis showed that prokinetic drugs are also effective for both of FD‐EPS and FD‐PDS. In Eastern countries, such as Japan in particular, that the therapeutic efficacy of prokinetic drugs on FD patients have been reported to be approximately twofold greater than in Western countries.^25^ Regardless of this, we did not note any additional effects when acotiamide was used in combination with PPI to the patients coexisting GERD and FD‐PDS, probably due to a scant involvement of gastric dysmotility in our patients.

Several studies have shown that acid infusion either into the stomach or into the duodenum led to the development of various symptoms of GERD and FD in both healthy subjects and FD patients.^26^, ^27^, ^28^ Our previous study also revealed that the therapeutic efficacy of PPI monotherapy in the patients with coexisting GERD and FD symptoms was greater in the group with more severe GERD symptoms. These results may indicate that FD symptoms in the patients coexisting with higher GERD symptoms are likely more acid‐related.^29^, ^30^

In conclusion, PPI monotherapy could be a proper initial treatment for patients coexisting with GERD and FD‐PDS.

Limitations: Some of the limitations of this study were that it was a single‐institution study with a retrospective design, the number of subjects was somewhat small, and that the association of the symptoms with reflux episodes or gastric pH not demonstrated, as pH‐impedance measurement was not conducted. Woefully, our sample size was somewhat scant; however, our results may provide the relevant information for the initial treatment of patients with coexisting GERD and FD‐PDS, as the treatment effect size was roughly the same for the groups. For future studies, a prospective, randomized controlled trial with a larger sample size is required.
